# Periarticular cooling reduces non-target perfusion in genicular artery embolization: a quantitative angiographic study

**DOI:** 10.1186/s42155-025-00597-0

**Published:** 2025-09-26

**Authors:** Alice M. Jacob, Alexander M. C. Böhner, Narine Mesropyan, Anna-Maria Odenthal, Alexander Isaak, Patrick Kupczyk, Daniel Kuetting, Julian Luetkens, Carsten Meyer, Tatjana Dell

**Affiliations:** Department of Diagnostic and Interventional Radiology and Quantitative Imaging Lab Bonn (QILaB), University Hospital Bonn, Venusberg-Campus 1, Bonn, 53127 Germany

**Keywords:** Genicular Artery Embolization (GAE), Transarterial Periarticular embolization (TAPE), Periarticular Cooling, Non-target Embolization, Quantitative Angiography, Cutaneous Complications, Knee Osteoarthritis, Safety, Technique

## Abstract

**Background:**

Genicular artery embolization (GAE) is an emerging, effective pain treatment for symptomatic knee osteoarthritis. A potential concern of this procedure is non-target embolization of cutaneous and subcutaneous vessels, which can lead to severe skin complications such as necrosis. To mitigate this risk, some operators use periarticular cooling with the rationale of inducing vasoconstriction. The vasoconstrictive effect on non-target subcutaneous vessels caused by cooling has, however, not yet been objectively demonstrated. This study aims to provide the first objective evidence for this common safety maneuver.

**Methodology:**

This retrospective analysis is based on a cohort of 36 patients (39 knees). The study evaluated a total of 49 selective angiographies of medial or lateral genicular arteries, which were stratified based on the presence of a superficial blush. Three cohorts were defined for comparison: 1) The Cooling Cohort, comprising 20 angiographies (from 10 knees) that showed a blush and were treated with periarticular cooling. 2) The Blush-Control Cohort, consisting of 18 angiographies (from 18 knees) with a blush but without a cooling maneuver. 3) The No-Blush Control Cohort (11 knees). The blush area on DSA was quantitatively analyzed for both groups with a superficial blush; in the Cooling Cohort, the area was compared before and after the intervention, while the baseline blush was quantified for the Blush-Control Cohort. Skin-related adverse events were systematically recorded and compared at the patient level across all three cohorts.

**Results:**

The application of periarticular cooling resulted in a significant reduction in the mean blush area from 464.8 ± 447.6 mm^2^ to 240.1 ± 208.2 mm^2^ (*p* = 0.012), corresponding to an average reduction of 73.8% (*p* = 0.0006). Patients receiving cooling showed significantly fewer skin alterations than the blush-control group [median score 1 vs 2, *p* = 0.0174].

**Conclusion:**

Periarticular cooling is a simple, non-invasive, and effective technique that significantly reduces quantifiable non-target cutaneous perfusion during GAE. Our work provides objective evidence supporting its use as a standard safety-enhancing maneuver to minimize the risk of skin-related complications.

## Background

Osteoarthritis (OA) is a significant global health issue, affecting around 595 million people and ranking as the third fastest-growing cause of disability [1]. For patients with chronic knee OA pain who have not responded to conventional treatments, genicular artery embolization (GAE) is a minimally invasive therapy that is gaining acceptance [2]. Also known as transarterial periarticular embolization (TAPE), GAE is being increasingly adopted in clinical practice as it has been shown to significantly improve function and quality of life, providing a new treatment option for this substantial patient population [3].

While GAE is generally considered a safe procedure, a recognized potential complication is the non-target embolization of cutaneous and subcutaneous arterial branches, which often originate from the genicular arterial network. This unintended deposition of particles can lead to a range of adverse skin events ranging from transient erythema and livedo reticularis to, in rare and severe cases, ischemic ulceration and necrosis, particularly when permanent embolic agents are used [2, 4, 5].

To mitigate this specific risk, many practitioners use periarticular cooling packs [4]. The rationale behind this is to induce localized vasoconstriction, which theoretically shunts blood flow away from superficial vessels and directs embolic particles more selectively towards the deep synovial tissue. However, this practice is largely based on empirical observation and physiological principles rather than robust scientific evidence. To date, the actual hemodynamic effect and clinical efficacy of this technique have not been objectively quantified during GAE.

Our work provides the first quantitative evidence of the effectiveness of this preventive maneuver. We used digital subtraction angiography (DSA) to objectively measure changes in cutaneous blush following cooling and followed up on patient outcomes.

## Methods

### Study design and patient population

This retrospective study was conducted in accordance with the Declaration of Helsinki and approved by the local institutional review board. All patients provided written informed consent for the GAE procedure and the anonymized use of their data for research purposes (Table 1).

**Table 1:** Patient demographics

	Blush-Control Cohort (*n* = 15 P; 18 K)	Cooling Cohort (*n* = 10 P; 10 K)	No-Blush Cohort (*n* = 11 P; 11 K)	*p*-value
Age (years, mean ± SD)	63.5 ± 16.6	64.7 6.5	72.3 ± 7.9	0.1841^a^
Gender (% male)	7/15 (47%)	5/10 (50%)	6/11 (55%)	0.925^b^
Kellgren-Lawrence Score (%)				0.647^c^
I	1/18 (5.6%)	0/10 (0%)	0/10 (0%)	
II	6/18 (33.3%)	2/10 (20%)	4/11 (36.4%)	
III	6/18 (33.3%)	5/10 (50%)	3/11 (27.3%)	
IV	5/18 (27.8%)	2/10 (20%)	2/11 (18.2%)	
Prosthetic Knee (%)	0/18 (0%)	1/10 (10%)	2/11 (18.2%)	0.298^b^

Data shown as n (%) or mean ± standard deviation

*P* Patients, *K* Knees

^a^One-way ANOVA

^b^Chi-square test

^c^Kruskal-Wallis test

We retrospectively screened all GAE procedures performed at our institution, initially identifying 83 procedures in 67 patients. For this focused analysis, a final cohort of 36 patients (39 knees) was selected based on the availability of complete angiographic documentation and clinical follow-up data. The primary indication for all patients was chronic knee pain (> 6 months) refractory to conservative therapies, with radiographic evidence of osteoarthritis (Kellgren-Lawrence grade I-IV).

To ensure an unambiguous correlation between a specific embolized vascular territory and potential skin changes, patients with the following characteristics were excluded from the analysis: 1) Significant superficial blush originating from both superior and inferior genicular arteries of the same compartment (e.g., medial superior and medial inferior). 2) Extensive collateral flow from the targeted vessel to the contralateral compartment (e.g., a medial genicular artery strongly supplying the lateral skin territory).

### GAE procedure and cooling technique

All procedures were performed by an experienced interventional radiologist. Under local anesthesia and ultrasound guidance, antegrade access to the ipsilateral common femoral artery was obtained, and a 5-Fr introducer sheath was placed. A 4-Fr diagnostic catheter was used for a baseline lower limb angiogram. Subsequently, selective catheterization of the target genicular arteries was performed using a 2.1-Fr microcatheter system.

Once the microcatheter was in position, a standardized baseline digital subtraction angiography (DSA) was acquired using a power-injected bolus of 10 mL of non-ionic contrast medium at 2.0 mL/s. For patients in the Cooling Cohort, a pre-frozen (−18 °C) gel pack was placed directly on the skin overlying the blush territory for exactly 5 min, followed by a second, identical DSA run. Therapeutic embolization for all patients was then performed using a suspension of Imipenem/Cilastatin.

### Angiographic evaluation and cohort stratification

The core analysis of this study was performed at the level of individual selective angiographies. A total of 49 such angiographies of medial or lateral genicular arteries were identified from the patient cohort and stratified into three groups for comparison:

1. The Cooling Cohort: This group included 20 selective angiographies from 10 patients (10 knees). These were characterized by a prominent superficial blush, for which the periarticular cooling technique was applied prior to embolization.
2. The Blush-Control Cohort: This group consisted of 18 selective angiographies from 15 patients (18 knees). These angiograms also demonstrated a significant blush, but embolization proceeded directly without a cooling maneuver.
3. The No-Blush Control Cohort: This cohort was formed by 11 patients (11 knees), in whom no significant superficial blush was detected during selective angiography.

### Quantitative angiographic analysis

A quantitative angiographic analysis was performed for all angiograms exhibiting a superficial blush (in the Cooling and Blush-Control cohorts) using Fiji software (Version 2.14.0/1.54f). For each case, a region of interest (ROI) was placed over the area of maximum blush on the baseline DSA. The area of contrast opacification (in mm^2^) within this ROI was quantified using a standardized densitometric threshold.

- In the Cooling Cohort, this quantification was performed on both the pre- and post-cooling DSA series to allow for a paired comparison and calculate the percentage reduction in blush area.
- In the Blush-Control Cohort, the same quantification was applied to the baseline DSA to serve as a control.

### Clinical follow-up

**Fig. 1:**
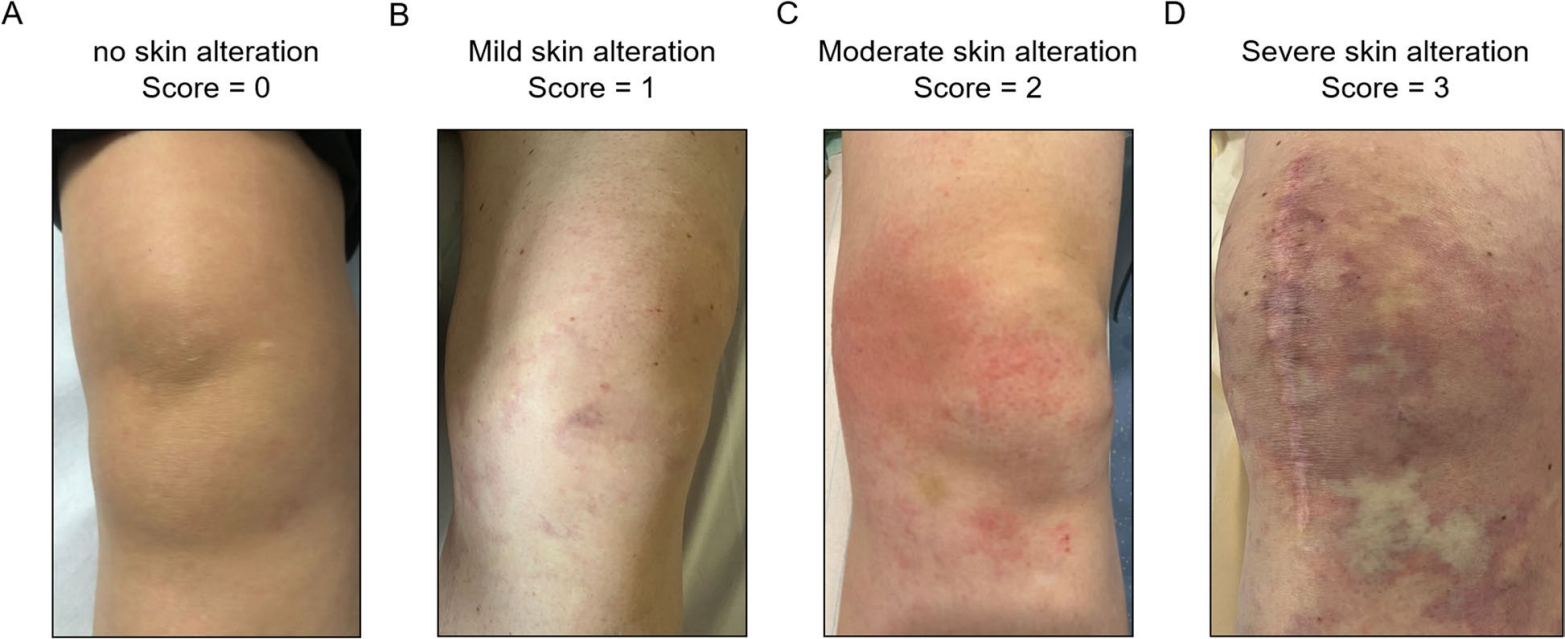
Skin alteration scores. **A** No skin alteration (Score 0). **B** Mild skin alteration (Score 1). **C** Moderate skin alteration (Score 2). **D** Severe skin alteration (Score 3)

Clinical follow-up was performed for all 36 patients across the three cohorts to compare outcomes. This included two key time points:

1. Immediate Post-procedural Assessment: The skin at the treatment site was examined immediately after the procedure. Standardized photographs of the treated area were taken to objectively document any adverse events, such as discoloration, erythema, or livedo reticularis. These findings were then classified according to a 4-point severity scale (Fig. 1).
2. 2-Week Follow-up: A second follow-up was conducted at 2 weeks, either in-person or via a standardized telephone interview, to specifically inquire about the delayed onset of skin blistering, ulceration, or necrosis.

### Statistical analysis

Statistical analyses were performed using GraphPad Prism (Version 10.4.0) and SPSS (Version 28.0), and a *p*-value of < 0.05 was considered statistically significant.

To assess the efficacy of the cooling maneuver within the Cooling Cohort (*n* = 20), the mean blush area before and after the intervention was compared using a paired-samples t-test. Continuous data were expressed as mean ± standard deviation (SD). For the comparison of baseline demographic and clinical characteristics across the three cohorts, a one-way ANOVA or the Kruskal–Wallis test was used for continuous variables, depending on data distribution. Categorical variables were compared using a Chi-square test.

To analyze the primary clinical outcome, the ordinal 4-point skin pathology scores were compared across the three cohorts using the Kruskal–Wallis test and pairwise comparisons were conducted using the Dunn’s multiple comparisons test to identify differences between specific groups.

## Results

### Patient cohort and angiographic characteristics

**Fig. 2:**
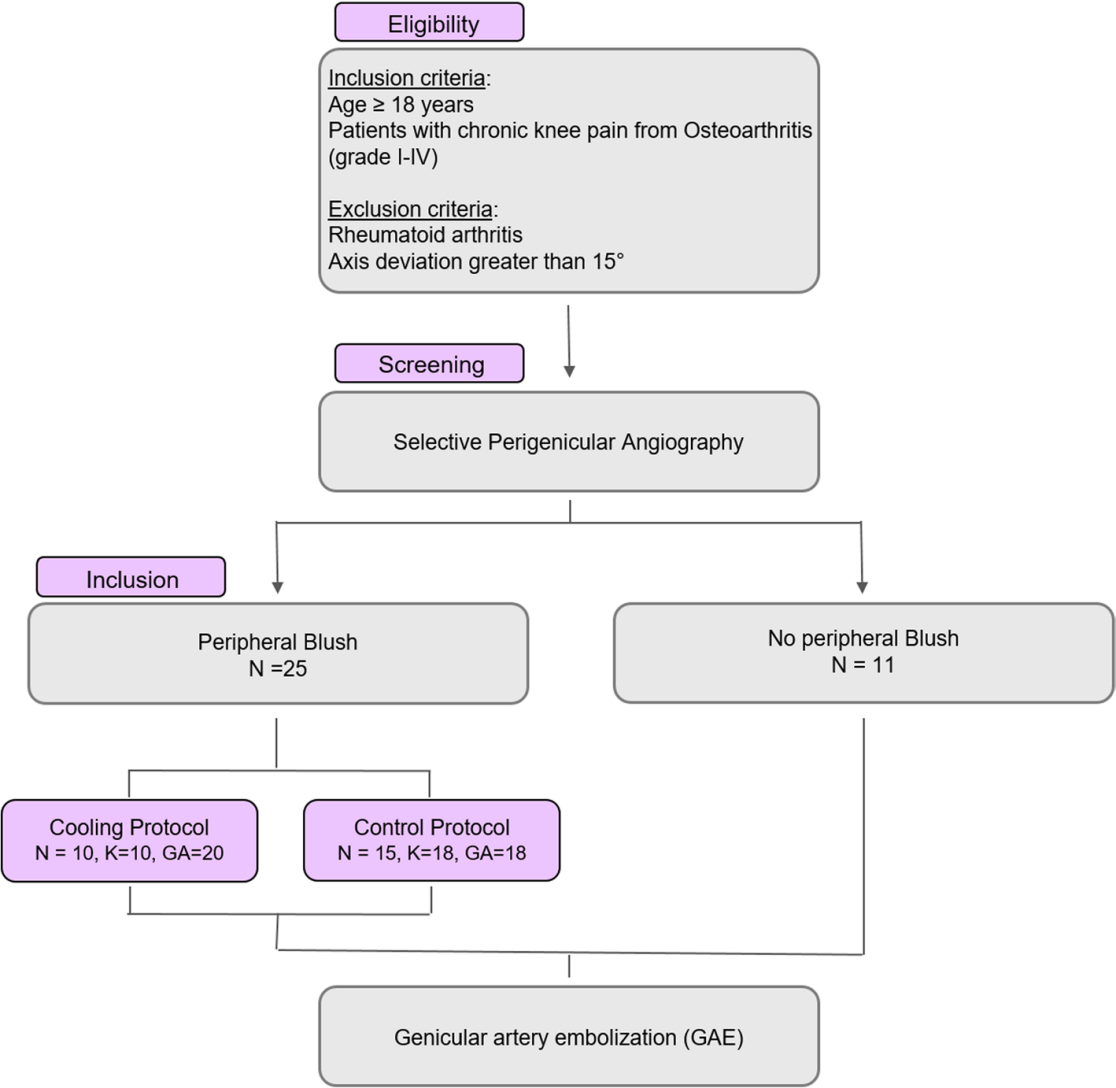
Flowchart of patient selection and cohort stratification. From an initial screening of 83 GAE procedures in 67 patients, a final cohort of 36 patients (39 knees) was included for analysis. The study is based on the evaluation of 49 selective genicular artery angiographies from this patient cohort, which were stratified based on the presence of a superficial blush and the subsequent treatment. Eleven patients (11 knees) without a blush formed the No-Blush Control Cohort. The remaining patients with a blush were allocated to either the Cooling Cohort (10 patients, 10 knees) or the Blush-Control Cohort (15 patients, 18 knees). The quantitative blush analysis was performed on the 20 angiographies from the Cooling Cohort and the 18 angiographies from the Blush-Control Cohort. Abbreviations: N, patients; K, knees

From an initial screening cohort of 67 patients who underwent 83 GAE procedures, a final study population of 36 patients (39 knees) met the inclusion criteria. From these patients, a total of 49 selective genicular artery angiographies formed the basis for the comparative analysis. Based on the presence of a superficial blush and the use of cooling, these were stratified into three cohorts (Fig. 2). A significant blush was observed in 25 patients, who were allocated to either the Cooling Cohort (10 patients; 10 knees) or the Blush-Control Cohort (15 patients; 18 knees). The remaining 11 patients (11 knees) without a blush formed the No-Blush Control Cohort.

### Baseline comparability and efficacy of periarticular cooling

**Fig. 3:**
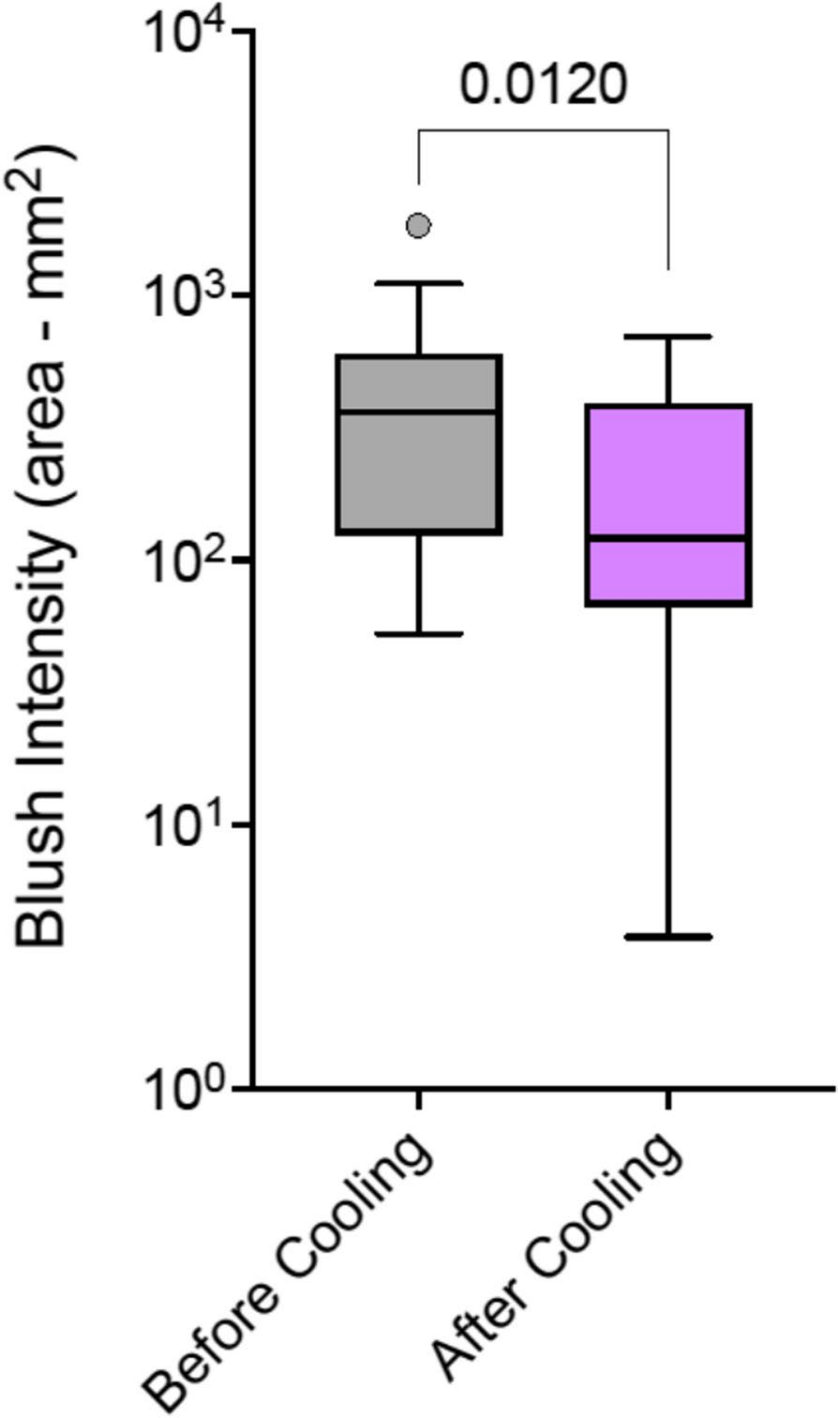
Peripheral Blush intensity before and after cooling. Blush intensity measured as the area covered by the blush before and after application of the cool pack. Box plot with Tukey whiskers; paired t-test

Quantitative analysis confirmed that there was no significant difference in the mean baseline blush area between the Cooling Cohort (464.8 ± 447.6 mm^2^) and the Blush-Control Cohort (651.3 ± 490.6 mm^2^, *p* = 0.2308), indicating a valid basis for comparison. The application of 5 min of periarticular cooling resulted in a profound and statistically significant reduction in non-target cutaneous perfusion. In the Cooling Cohort, the mean blush area decreased from 464.8 ± 447.6 mm^2^ to 240.1 ± 208.2 mm^2^ (*p* = 0.012), corresponding to a mean blush reduction of 73.8% (*p* = 0.0006) (Fig. 3).

### Clinical outcomes and skin-related adverse events

**Fig. 4:**
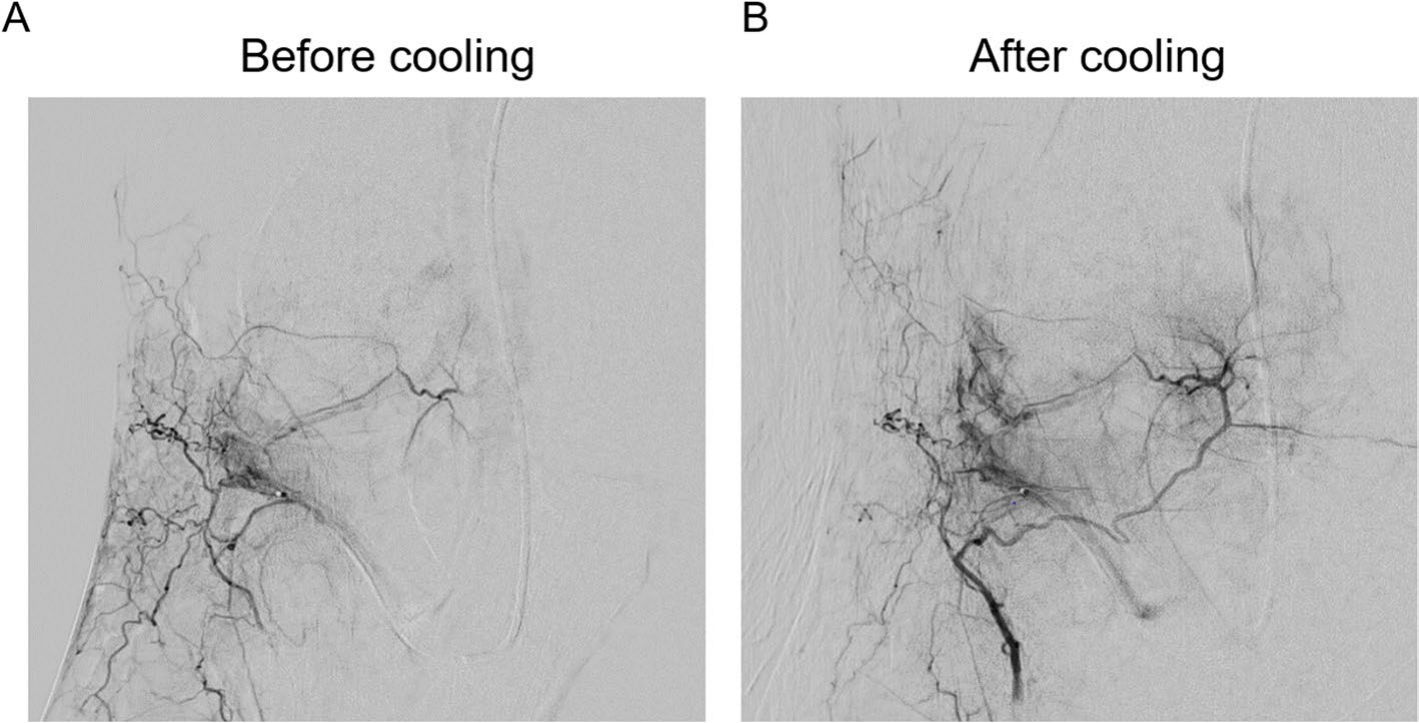
Angiographic effect of periarticular cooling. **A** Baseline digital subtraction angiography (DSA). A 4F diagnostic catheter is positioned at the ostium of the lateral inferior genicular artery, with a microcatheter advanced distally to the target embolization site. The angiogram demonstrates a prominent superficial, non-target blush and collateral filling of the recurrent anterior tibial artery. **B** Corresponding DSA after 5 min of periarticular cooling reveals a marked reduction of the superficial blush. The induced vasoconstriction redirects perfusion, leading to a more profound and targeted penetration of contrast medium into the deep synovial enhancement

Immediate post-procedural assessment revealed only transient, mild erythema in a majority of patients, all of which resolved within hours. No cases of livedo reticularis, blistering, ulceration, or skin necrosis were observed at the 2-week follow-up in any patient. A comparison of skin pathology scores revealed a statistically significant difference among the three groups (Kruskal–Wallis test, *p* = 0.035). Post-hoc analysis demonstrated that patients in the Cooling Cohort had significantly lower skin pathology scores than those in the Blush-Control Cohort (median score 1 vs. 2, *p* = 0.0174). The scores in the Cooling Cohort were statistically indistinguishable from those in the No-Blush Control Cohort (median score 1 vs. 1, *p* > 0.9999) (Fig. 4).

## Discussion

The prominence of genicular artery embolization (GAE) as an emerging pain therapy for symptomatic knee osteoarthritis (OA) has grown substantially in recent years, both in scientific literature and clinical practice. It offers a valuable alternative for patients with medically refractory disease and for those who are not eligible for, or do not wish to undergo, total knee arthroplasty [2]. However, this rapid adoption has outpaced the standardization of the procedure, leaving many open questions regarding optimal technique, patient selection, and outcome measurement. Consequently, professional bodies like the Society of Interventional Radiology (SIR) have called for a more homogenous approach and have published statements to help standardize the practice [5].

The recent reporting SIR standards explicitly recommend the use of cooling [5], albeit based on expert consensus rather than quantitative data. Similarly, pioneers in the field such as Okuno et al. have described the technique in their case series but never objectively measured its hemodynamic effect [6]. Our study closes this crucial evidence gap by transforming an anecdotal recommendation into an evidence-based practice and quantifying the physiological effect for the first time. This is particularly relevant as complication rates involving skin necrosis, although rare, have been documented in the literature with the use of permanent particles [7].

We provide the first quantitative evidence that periarticular cooling profoundly reduces non-target perfusion during GAE, demonstrating a mean blush area reduction of 73.8%. The physiological basis for this finding lies in the direct effect of cold on cutaneous arterioles. Local hypothermia stimulates alpha-adrenergic receptors in vascular smooth muscle cells, leading to potent vasoconstriction and explaining the significant angiographic changes we observed [8]. When compared to other strategies for preventing non-target embolization, such as meticulous super selective catheterization or using larger particles (which may limit therapeutic efficacy), periarticular cooling offers the distinct advantages of being simple, non-invasive, and universally applicable without systemic side effects.

Beyond its role in mitigating cutaneous complications, our study suggests a potential dual benefit of periarticular cooling. By inducing vasoconstriction in superficial, non-target vessels, the maneuver appears to shunt arterial flow more effectively toward the deeper, inflamed synovial tissue. This redirection resulted in a visibly enhanced and more concentrated synovial blush on angiography. This finding implies that cooling could serve not only as a safety adjunct for permanent embolic agents but also as an efficacy-enhancing tool. By optimizing the delivery of the therapeutic agent to the site of pathology, it may improve the technical outcome of the embolization.

This study has several limitations that must be acknowledged. First, its retrospective design and relatively small sample size, particularly with the Cooling Cohort comprising only 10 knees, may limit the generalizability of the findings. While achieving statistical significance even with this limited sample size suggests a robust treatment effect, we acknowledge that these promising results require validation in larger, prospective trials. The cooling intervention was not applied in a randomized fashion but was based on the operator's judgment, introducing a potential selection bias. Since this decision was not based on pre-defined, controlled criteria, it cannot be ruled out that systematic differences between the Cooling and the Blush-Control cohorts influenced the outcome beyond the cooling maneuver itself. Second, a limitation relates to the complex vascular anatomy of the knee. Although we implemented strict exclusion criteria for obvious cross-compartmental collateralization, the knee's richly collateralized synovial plexus means that subtle, residual cross-perfusion cannot be entirely ruled out. Consequently, this could potentially confound the direct attribution of a specific skin alteration to the embolized vascular territory. Third, our clinical follow–up was limited to two weeks. While this timeframe is clinically appropriate for detecting the onset of severe cutaneous complications like necrosis, it is insufficient to assess the long-term clinical efficacy of the procedure. Future studies with extended follow-up are warranted to investigate whether the enhanced synovial perfusion observed after cooling translates into a more durable improvement in pain and function. Fourth, the 4-point severity scale used for assessing skin alterations was not externally validated. This scale was developed to ensure systematic and consistent documentation in the absence of a standardized assessment tool for skin changes post-GAE. To minimize potential observer variability, all assessments were supported by standardized photographic documentation. We acknowledge, however, that the development of a consensus-based, validated scoring system is an important next step for the field to ensure comparable and objective reporting of outcomes in future studies. Finally, this study exclusively utilized Imipenem/Cilastatin, a temporary embolic agent. However, the core finding of our work is the demonstration of a physiological response – vasoconstriction – which is inherently independent of the embolic material used. Our study thus provides a technical proof-of-concept that is highly relevant for all types of GAE, particularly when using permanent microspheres, where the risk of cutaneous complications is significantly higher. We therefore propose that our findings provide a strong rationale for implementing periarticular cooling as a fundamental, agent-agnostic safety maneuver to be considered in all GAE procedures.

## Conclusion

This study provides the first objective, quantitative evidence that periarticular cooling is a highly effective technique for significantly reducing non-target cutaneous perfusion during GAE. It transforms a practice based on anecdotal experience into an evidence-supported safety measure. Given its simplicity and effectiveness, our findings support the routine implementation of periarticular cooling as a standard, safety-enhancing maneuver to minimize the risk of skin-related complications and improve the overall safety of this promising therapy.

## Data Availability

The datasets used and/or analyzed during the current study are available from the corresponding author on reasonable request.

## Abbreviations

GAE: Genicular Artery Embolization
TAPE: Transarterial Particle embolization
OA: Osteoarthritis
SIR: Society of Interventional Radiology
